# Effectiveness of en masse versus two-step retraction: a systematic review and meta-analysis

**DOI:** 10.1186/s40510-017-0196-7

**Published:** 2018-01-05

**Authors:** Mumen Z. Rizk, Hisham Mohammed, Omar Ismael, David R. Bearn

**Affiliations:** School of Dentistry, University of Dundee, Nethergate, Dundee, DD1 4HN UK

**Keywords:** Space closure, Orthodontic anchorage procedures, Root resorption, Canine retraction, En masse retraction, Systematic review, Meta-analysis

## Abstract

**Background:**

This review aims to compare the effectiveness of en masse and two-step retraction methods during orthodontic space closure regarding anchorage preservation and anterior segment retraction and to assess their effect on the duration of treatment and root resorption.

**Methods:**

An electronic search for potentially eligible randomized controlled trials and prospective controlled trials was performed in five electronic databases up to July 2017. The process of study selection, data extraction, and quality assessment was performed by two reviewers independently. A narrative review is presented in addition to a quantitative synthesis of the pooled results where possible. The Cochrane risk of bias tool and the Newcastle-Ottawa Scale were used for the methodological quality assessment of the included studies.

**Results:**

Eight studies were included in the qualitative synthesis in this review. Four studies were included in the quantitative synthesis. En masse/miniscrew combination showed a statistically significant standard mean difference regarding anchorage preservation − 2.55 mm (95% CI − 2.99 to − 2.11) and the amount of upper incisor retraction − 0.38 mm (95% CI − 0.70 to − 0.06) when compared to a two-step/conventional anchorage combination. Qualitative synthesis suggested that en masse retraction requires less time than two-step retraction with no difference in the amount of root resorption.

**Conclusions:**

Both en masse and two-step retraction methods are effective during the space closure phase. The en masse/miniscrew combination is superior to the two-step/conventional anchorage combination with regard to anchorage preservation and amount of retraction. Limited evidence suggests that anchorage reinforcement with a headgear produces similar results with both retraction methods. Limited evidence also suggests that en masse retraction may require less time and that no significant differences exist in the amount of root resorption between the two methods.

**Electronic supplementary material:**

The online version of this article (10.1186/s40510-017-0196-7) contains supplementary material, which is available to authorized users.

## Background

Tooth extraction for orthodontic purposes has been a controversial topic for the past century [1–3]. This conflict is still brewing among orthodontists nowadays. Modern practitioners seem to have reached a middle ground when it comes to the decision to extract or not to extract [4, 5]. Space closure is one of the main stages of orthodontic treatment when extractions are undertaken as part of the treatment plan. It is a complicated multifactorial process that requires knowledge, skill, and experience to complete successfully [6]. Space closure can be achieved using one of the two methods, either sliding mechanics (frictional mechanics) or closing loops (frictionless mechanics).

The use of those two methods depends mainly on the treatment plan, appliance used, and the clinician’s preference. Closing loops were mostly used for space closure with standard edgewise appliances [7, 8] due to the presence of archwire bends (i.e., first-, second-, and third-order bends) which made the use of any other method of space closure impossible [9]. The introduction of the pre-adjusted edgewise appliance by Andrews eliminated the need for these bends giving rise to what is known as the straight-wire technique [10] which allows for the use of sliding mechanics requiring movement between the archwire and the bracket, which is resisted by friction, binding and then notching [11]. Space closure using sliding mechanics can be achieved either by separately retracting the canine followed by the four incisors (two-step) or by en masse retraction of the whole anterior segment simultaneously [12]. Some claim that the two-step technique produces less strain on the anchor unit. In theory, the division of the active unit into canines followed by the four incisors should result in less anchorage loss. These claims are based on the difference in the periodontal ligament surface area between the active unit and the anchor unit at all times [6]. On the other hand, this is seen as a complicated and time-consuming technique by some practitioners who claim that dividing up the strain does not negate its overall effect on the anchor unit. The choice of either of these techniques depends on the clinician’s experience and preference [13]. In clinical practice, the clinician is looking for space closure mechanics that provide good anchorage control and less treatment time. Unfortunately, there have been limited attempts to compare between the two main space closure methods in literature and a systematic review comparing en masse and two-step retraction has not been undertaken. This review aims to compare between en masse retraction and two-step retraction evaluating their effect on the amount of anchorage loss and amount of anterior retraction during space closure. This review will also explore the difference between the two methods regarding treatment time and root resorption.

## Methods

### Protocol and registration

The protocol was not published online. This review followed the guidelines of the Preferred Reporting Items for Systematic Reviews and Meta-Analyses (PRISMA).

### Eligibility criteria

Study design: Randomized controlled trials (RCTs) and prospective controlled clinical trials (pCCTs).

Participants: Orthodontic patients treated with pre-adjusted fixed appliances requiring space closure in the maxillary arch.

Intervention: En masse retraction method to achieve space closure.

Comparison: Two-step retraction method to achieve space closure.

Primary outcomes: Anchorage loss and the amount of incisor retraction.

Secondary outcomes: Duration of treatment/retraction and the amount of root resorption.

These outcomes are reported using measurements from lateral cephalometric X-rays, other two-dimensional X-rays, or three-dimensional radiographic analysis.

Exclusion criteria: Retrospective design studies, case reports, studies using lingual fixed appliances, and secondary studies.

### Sources, search strategy, and study selection

The electronic database search was performed up to July 2017 independently by two reviewers in five electronic databases (MEDLINE, Scopus, Web of Science, PubMed, and the Cochrane Central Register of Controlled Trials (CENTRAL)) with no initial restriction on language, publication dates, or study designs. An additional screening of the reference lists of potentially eligible articles was conducted. The assessment of risk of bias and data extraction was performed independently by two reviewers. Authors were contacted in case there was any missing information. Any disagreements between the two reviewers were discussed and resolved with a third reviewer. The terms used are shown in Additional file 1: Table S1.

### Data extraction

Data extraction of the included studies was performed independently by two reviewers using customized data extraction forms. The data extraction form included study identification, publication date, article title, study design, study location, funding, sample size, age and sex, the diagnosis of the malocclusion, type of anchorage reinforcement, method of space closure, measurement method and time, type of force delivery system, type of archwire used, type of fixed appliance, dental changes, duration of space closure, and amount of root resorption. Authors were contacted in case of any missing information.

### Risk of bias/quality assessment

Two reviewers assessed the quality of eligible studies independently. The Cochrane risk of bias tool [14] was used to assess the methodological quality of the RCTs. The studies were assessed to be of low, high, or unclear risk of bias based on seven domains [random sequence generation, allocation concealment, blinding of participants and personnel, blinding of outcome assessment, incomplete outcome data, selective reporting, and other bias]. If one of the domains was assessed to be of high risk of bias, the study was given an overall score of high risk. The Newcastle-Ottawa Scale was used to assess the quality of the non-randomized prospective controlled trials [15]. The studies were awarded stars according to how well they were designed. A maximum of one star can be awarded for each of the seven items within the selection and outcome categories. In the comparability category, a maximum of two stars can be awarded. The highest score that can be awarded to a single study is nine stars. Studies scoring less than six stars are considered to be of low quality. On the other hand, a score of more than six stars imply high-quality design. Any differences in judgment were resolved with a third reviewer.

### Summary measures and synthesis of the results

Data from the included studies was summarized according to each outcome of interest. An attempt was made to pool the results of studies reporting on the same outcome, measured at the same time points, and using the same method of measurement. Data synthesis was performed using the Review Manager (RevMan) (version 5.3. Copenhagen: The Nordic Cochrane Centre, the Cochrane Collaboration). For continuous outcomes, reported means, standard deviations, and sample sizes were utilized to combine the results into a standardized mean with a 95% calculated confidence interval accounting for possible differences in the measurement points. In the case of failure to combine any of the included studies, a narrative synthesis was performed. A random-effects model was used in anticipation of any possible heterogeneity. *I*^2^ test was used to evaluate the magnitude of existing heterogeneity where a score from 0 to 30% is considered low and more than 50% is considered to be of high heterogeneity [16]. Tau-squared test was used to indicate any presence of heterogeneity. Furthermore *P* value was used to identify the existence of significant heterogeneity where (*P* < 0.1) means significant heterogeneity.

### Additional analyses

Sensitivity analysis was performed in an attempt to detect the sound methodological approaches of including pCCTs and to isolate the impact of studies with high risk of bias. This also helped in detecting the effect of individual studies on the overall result. Publication bias was identified through the inspection of the generated funnel plots in the case of the inclusion of more than 10 studies.

## Results

### Study selection and characteristics

**Fig. 1:**
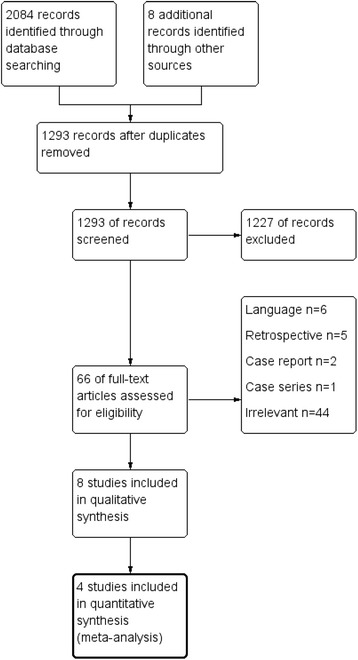
Flow diagram of the literature search

The electronic search yielded a total of 2084 studies. Eight more studies were identified by hand searching the reference lists of eligible articles. The titles and abstracts of 1293 studies were screened after duplicates were removed. The full text of 66 studies was assessed for eligibility against the inclusion and exclusion criteria. Fifty-eight studies were excluded with reasons. Eight studies were found to be compatible with the inclusion criteria and were included in this review. The study selection process is shown in the flow chart (Fig. 1). The eight remaining studies included four RCTs and four pCCTs (Table 1). These studies were divided into four different comparison groups according to the type of anchorage reinforcement used in each group during space closure (Table 2).

**Table 1:** Characteristics of the included studies

Study info.	Study design	G	Sample no.	Age	Gender	Malocclusion	Archwire/bracket	Anchorage and Aux	Force system/amount	Measurement *T*
Al-Sibaie and Hajeer 2013 [18]	RCT	G1	28	23.02	19F/9M	Class II div. 1/OJ > 5 Crowding <3.5	0.019 × 0.025″ SS/0.022″ MBT	Miniscrews	Elastic-chain 150 g	T1 before ttt/T2 after leveling/T3 class I canine
		G2	28	20.46	16F/12M		0.019 × 0.025″ SS/0.022″ MBT	TPAs	Elastic-chain	
Davoody et al. 2012 [20]	RCT	G1	23	17.4 SD 8.8	6F/7M	Angle class I or II	0.016 × 0.022″ SS/0.022″ slot SL	Miniscrews	NiTi coil 150 g	T1 before retraction
		G2	23	17.9 SD 8.9	10F/5M		0.016 × 0.022″ SS/0.022″ slot SL	Differential moment	NiTi coil 150 g (canine) then 0.017 × 0.025 SS loop (incisors) and 0.017 × 0.025 NiTi intrusion arch	T2 after retraction
Huang et al. 2010 [24]	Prospective	G1	26	15.4 SD 1.9	18F/8M	Class I or II	0.018 × 0.025″ SS/0.022″ slot		NiTi coil 150 g	T1 before space closure
		G2	26	15.8 SD 1.8	18F/8M		0.018 × 0.025″ SS/0.022″ slot		NiTi coil 150 g (canine) then (incisors)	T2 after space closure
Kuroda et al. 2009 [23]	Prospective	G1	11	18.5 SD 3.3	F	Class II/ OJ > 5		Miniscrews	NiTi coil 100 g	T1 before ttt
		G2	11	21.8 SD 7.9	F			TPA and HG	Sliding mechanics (canine) then loop mechanics (incisors)	T2 after ttt
Solem et al. 2013 [22]	Prospective	G1	11	20 to 29 mean 24 years	20F/4M	Angle class I/ severe dentoalveolar protrusion	0.016 × 0.022″ SS/0.018″ slot	Miniplates	Elastic-chain	T1 before ttt
		G2	13				0.016 × 0.022″ SS/0.018″ slot	TPAs	Elastic-chain (canine) then intrusion retraction loop (incisors)	T2 after ttt
Upadhyay et al. 2008 [21]	Prospective	G1	15	14.5 to 22.3 mean 17.2	10F/5M	Angle class I/ class II div. 1 with severe OJ	0.017 × 0.025″ SS/0.022″ ROTH	Miniscrews	NiTi coil 150 g	T1 before retraction
		G2	15		11F/4M			Nance, HG, Bond7		T2 after retraction
Upadhyay et al. 2008 [17]	RCT	G1	20	17.6years	F	OJ = <5/Crowding < 3.5	0.017 × 0.025″ SS/0.022″ ROTH	Miniscrews	NiTi coil 150 g	T1 before retraction
		G2	20	17.3years	F		0.017 × 0.025″ SS/0.022″ ROTH	TPA, HG, Bond7		T2 after space closure
Xu et al. 2010 [19]	RCT	G1	32	12.6 SD 1.1	20F/12M	Class I or II	0.022″ MBT	32 HG/27 TPA	Active laceback	T1 before ttt
		G2	32	12.7 SD 1.2	19F/12M		0.022″ MBT	31 HG/21 TPA	Active laceback	T2 after ttt

*G* group, *Aux* auxiliaries, *T* time, *RCT* randomized controlled trial, *OJ* overjet, *ttt* treatment, *SS* stainless steel, *SL* self-ligation, *HG* headgear, *TPA* transpalatal arch

**Table 2:** The different comparison groups

Comparison no.	Space closure method	Anchorage reinforcement	VS	Space closure method	Anchorage reinforcement	Study included	Anchorage classification
Comparison 1	En masse retraction	Miniscrews	VS	Two-step retraction	Headgear	Kuroda et al. 2009 [23]	Maximum anchorage
Comparison 2	En masse retraction	Miniscrews	VS	Two-step retraction	Conventional anchorage	Al-Sibaie and Hajeer 2013 [18]	Maximum anchorage
						Davoody et al. 2012 [20]	Maximum anchorage
						Solem et al. 2013 [22]	Moderate anchorage
						Upadhyay et al. 2008 [17]	Maximum anchorage
						Upadhyay et al. 2008 [21]	Maximum anchorage
Comparison 3	En masse retraction	Headgear	VS	Two-step retraction	Headgear	Xu et al. 2010 [19]	Maximum anchorage
Comparison 4	En masse retraction	Conventional anchorage	VS	Two-step retraction	Conventional anchorage	Huang et al. 2010 [24]	Moderate anchorage

### Risk of bias within included studies

**Fig. 2:**
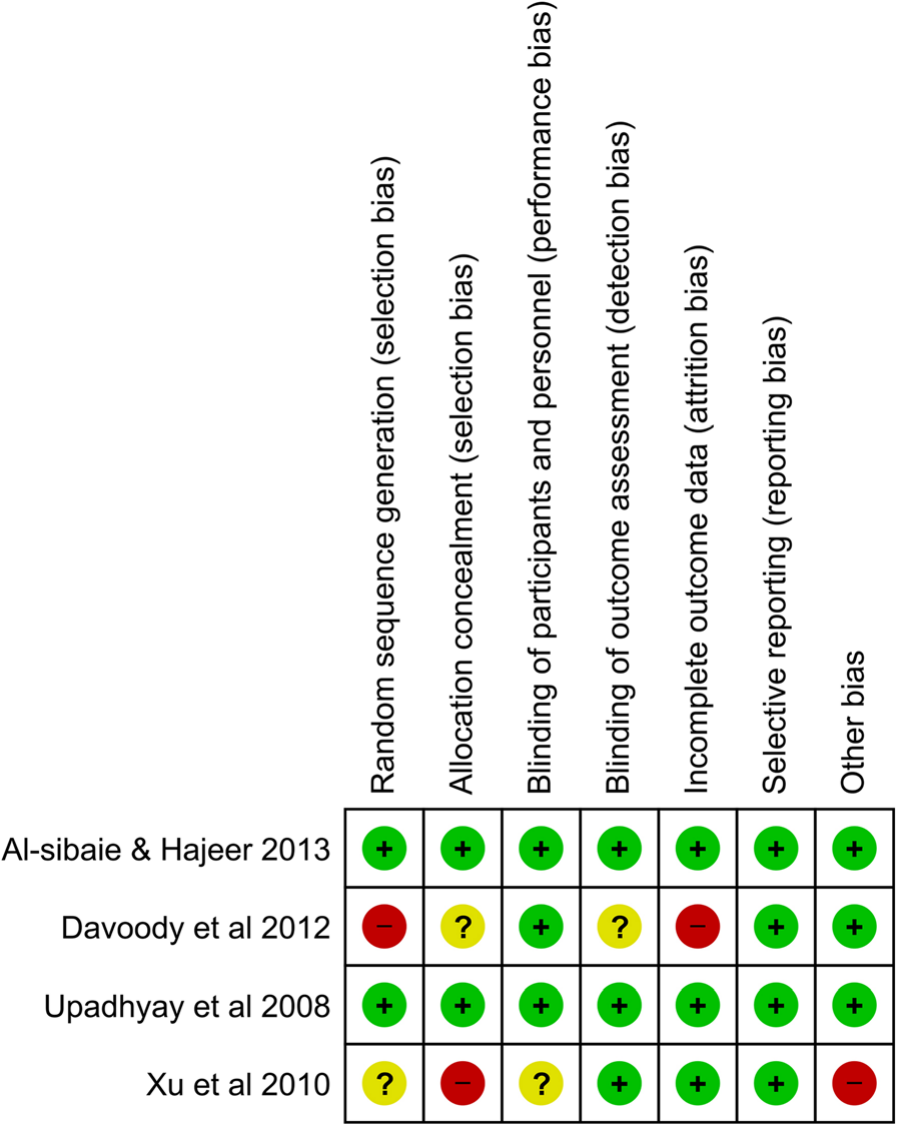
Risk of bias for randomized controlled trials. Low risk of bias (green). Unclear risk of bias (yellow). High risk of bias (red)

Two RCTs [17, 18] were found to be of low risk of bias while the other two studies [19, 20] were assessed to be of high risk of bias. Performance bias was unavoidable in this instance as the operators were directly involved in the interventions so the judgment was lenient yet objective due to the impossibility of blinding. Allocation concealment was not mentioned in two of the RCTs, and both authors were contacted. One author responded and provided the needed information [17] while the other [19] did not respond. The results of the quality assessment for RCTs are shown in Fig. 2.

**Fig. 3:**
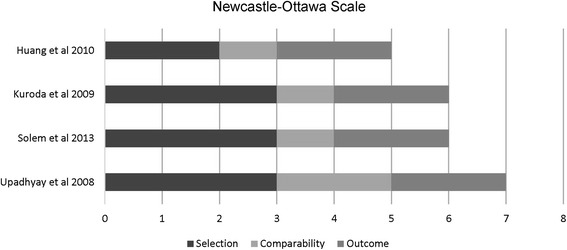
Quality assessment of the prospective non-randomized trials

Four non-randomized prospective trials were assessed using the Newcastle-Ottawa Scale. Three studies were assessed to be of high quality [21–23] while one study [24] was assessed to be of low quality. The results of the quality assessment for non-randomized prospective trials are shown in Fig. 3.

### Results of individual studies, synthesis, and additional analyses

#### Comparison group 1 (en masse with miniscrews versus two-step with headgear)

One study [23] was identified in this group.

##### Anterio-posterior movement of the upper central incisors in millimeters

The difference in the amount of the distal movement of the maxillary central incisors (UI) was reported to be significant (*P* < 0.01) between the two methods. A greater amount of incisors retraction was achieved in the en masse/miniscrew group (− 9.3 mm, SD 2.03) compared to the two-step/headgear group (− 6.3 mm, SD 1.44).

##### Anterio-posterior movement of the upper first molars in millimeters

The difference in the amount of mesial movement of the upper first molars (U6) was reported to be significant (*P <* 0.01) between the two retraction methods. An average of 3 mm of mesial movement of the U6 was reported in the two-step/headgear group in contrast to minimal movement reported in the en masse/miniscrew group (0.7 mm, SD 0.64).

#### Comparison group 2 (en masse with miniscrews versus two-step with conventional anchorage)

Five studies [17, 18, 20–22] were identified in this group.

##### Anterio-posterior movement of the upper central incisors in millimeters:

**Fig. 4:**
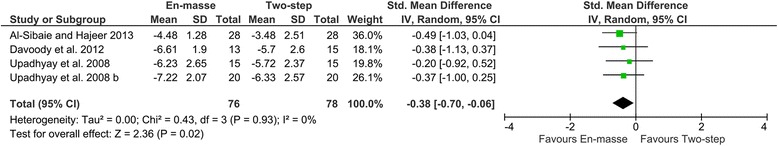
Forest plot showing the amount of retraction with random-effects model and 95% CI

Al-Sibaie and Hajeer [18] reported a significant difference in the amount of UI movement between the two groups with more retraction in the en masse group. The remaining four studies found no significant difference in the distal movement of the UI between the two retraction methods. A greater distal movement of the UI was detected in the en masse/miniscrew group with a standardized (std) difference in means of − 0.38 mm (95% CI − 0.70 to − 0.06) between the two groups. The difference in the amount of incisor retraction represented by the distal movement of the UI was found to be statistically significant between the two retraction methods (*P* < 0.05) (Fig. 4).

##### Anterio-posterior movement of the upper first molars in millimeters:

**Fig. 5:**
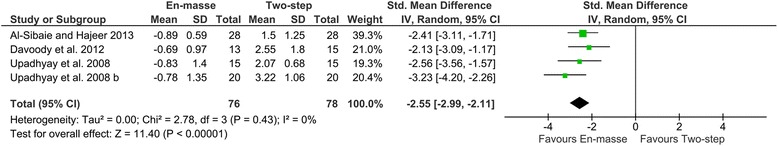
Forest plot showing the amount of anchorage loss with random-effects model and 95% CI

The posttreatment position of the U6 was reported to be distal to its original position in the en masse group. A wide range of values of the mesial movement of the U6 was reported in the two-step group (1.50 to 3.22 mm). A greater mesial movement of the U6 was detected in the two-step/conventional anchorage group with a std difference in means of − 2.55 mm (95% CI − 2.99 to − 2.11) between the two methods. The difference in the amount of anchorage loss represented by the mesial movement of the U6 between the two retraction methods was found to be statistically significant (*P* < 0.001) (Fig. 5).

Four of the five studies were combined in the meta-analyses. A different measurement tool was used by the fifth study [22] making its inclusion in the quantitative analyses not possible.

##### Duration of retraction/treatment

Three studies reported either the duration of retraction or the overall treatment. No significant difference was reported in the duration of retraction between the two methods in two studies [17, 21] in contrast to Al-Sibaie and Hajeer [18] who reported 4.7 more months in the two-step/conventional group with a statistically significant difference in the overall treatment time between the two groups.

#### Comparison group 3 (en masse with headgear versus two-step with headgear)

One study [19] was identified in this group.

##### Anterio-posterior movement of the upper central incisors in millimeters

No significant difference was reported in the amount of incisor retraction between the two groups. The distal movement of the UI was reported to be 5.7 mm (SD 2.4) and 5.7 mm (SD 2.0) in the two-step/headgear group and en masse/headgear group respectively.

##### Anterio-posterior movement of the upper first molars in millimeters

Less mesial movement of the U6 in was reported in the en masse group (4.1 mm, SD 2.0), yet no significant difference was found between the two groups. In the two-step/headgear group, mesial movement of the U6 was reported to be (4.5 mm, SD 2.2).

##### Duration of treatment

No significant difference was reported in the duration of treatment between the two-step group (2.6 years, SD 0.8) and the en masse group (2.5 years, SD 0.9).

##### Apical root resorption (RR)

No significant difference was reported in the amount of root resorption between the two groups.

#### Comparison group 4 (en masse with conventional anchorage versus two-step with conventional anchorage)

One study [24] was identified in this group.

##### Duration of space closure

Space closure required lesser time to be achieved in the en masse group (5.8 months, SD 1.4) compared to the two-step group (7.9 months, SD 1.8) with a significant difference in the duration of space closure (*P* < 0.001).

##### Apical root resorption (RR)

Maxillary central incisors: Reported no significant difference in the amount of root resorption between the two-step group (0.45 mm, SD 0.13) and the en masse group (0.42 mm, SD 0.12).

Maxillary lateral incisors: Reported no significant difference in the amount of root resorption between the two-step group (0.60 mm, SD 0.11) and the en masse group (0.56 mm, SD 0.08).

### Risk of bias across studies

No test was undertaken as less than 10 studies were included in the meta-analysis.

### Sensitivity analysis

**Fig. 6:**
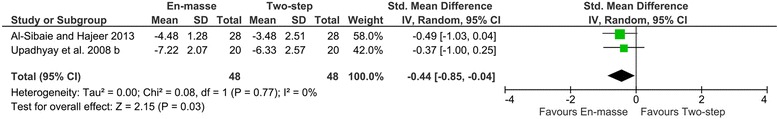
Forest plot showing sensitivity test for the amount of retraction of the UI

**Fig. 7:**
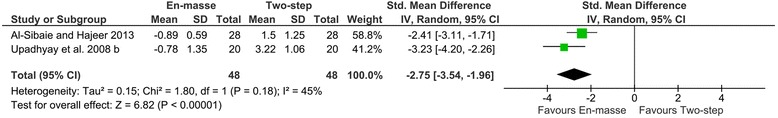
Forest plot showing sensitivity test for the amount of anchorage loss in the U6

RCTs with potentially high risk of bias and non-randomized prospective trials were excluded from the meta-analysis. This was to test the impact of individual studies on the overall results. The removal of these low-quality studies increased the confidence in the results. The heterogeneity was assessed using *I*-squared, Tau-squared, and chi-squared tests (Figs. 6 and 7).

## Discussion

Orthodontic treatment planning revolves around attaining Andrews’ six keys of occlusion [25]. Ending up with stable class I incisor, canine and molar relationships are the goal of any orthodontist. Achieving these goals in extraction cases is an expression of the orthodontist’s understanding of the integral relationship between space closure mechanics and the anchorage required. The orthodontic treatment plan for extraction cases is based on the individual needs of every single patient. Selecting the suitable space closure mechanics for the right case is paramount for the success of the treatment.

### Anchorage loss:

Seven out of eight included studies reported on both the amount of anchorage loss and amount of incisor retraction. Those seven studies reported a significant difference in the amount of anchorage loss between the two methods of retraction. Less mesial movement of the anchor unit was reported in the en masse retraction group regardless of the anchorage method except for one study [19]. They reported no significant difference in the amount of anchorage loss. Comparison group 1 reported a very small amount of mesial movement of the U6 in the en masse group (0.7 mm). They speculated that this movement occurred during the leveling and alignment phase as the miniscrews were not inserted till after the phase was over. During that period of time, the U6 was free to migrate in the mesial direction. Their findings were consistent with the other studies [26–28] who all reported a similar minimal mesial movement of the U6. The mesial movement of the U6 reported by these studies is in contrast with the findings of comparison group 2 [17, 18]. They reported no mesial movement of the U6; moreover, an actual anchorage gain occurred. Some authors [17, 18] attributed that the action of the NiTi closing coils which was left in place after contact between the canine and the posterior segment was achieved. This continued action after achieving contact might have translated the forces to the U6 through the interdental contacts causing a distalizing force on the U6. Davoody and colleagues [20] speculated that as the wire moves through the tube of the U6 during en masse retraction, the friction between the wire and the tube exerts a distal force on the U6 causing it to move distally.

Data synthesis in comparison group 2 showed a statistically significant difference in the amount of anchorage loss with a standard mean difference of − 2.55 mm. These results show less anchorage loss in the en masse/miniscrew group when compared to the two-step retraction combined with conventional anchorage. This amount of anchorage loss is considered to be clinically significant as 2.5 mm of the extraction space can greatly impact the treatment outcomes. The two-step retraction is claimed to preserve anchorage. This claim is the reason behind combining it with conventional anchorage techniques. Both en masse/miniscrew combination and two-step retraction combined with a conventional anchorage are used in maximum anchorage cases. In a clinical setting, this should not be considered a comparison between two retraction mechanics or anchorage methods; rather, it is between two maximum anchorage protocols. These results suggest that in maximum anchorage extraction cases, the use of two-step/conventional anchorage combination is less effective in preserving anchorage. Keeping in mind the individuality of each case, the clinician should always strive to use the most effective method. The two-step retraction method could still be used where there is a need to retract the canines first to allow for the alignment of the incisors; however, according to these findings, the use of an en masse/miniscrew protocol is more effective in anchorage preservation in maximum anchorage cases. These findings are consistent with recent meta-analyses which conclude that the use of en masse/miniscrew combination provides better anchorage control than conventional methods of anchorage [29].

Comparison group 3 reported no significant difference in the amount of anchorage loss between the two retraction methods. A difference of − 0.36 mm mesial movement of the U6 was reported between the two groups. Less mesial movement of the U6 was reported in the en masse group, yet the authors mentioned that this was unlikely to be representative of the population. They added that these results were probably due to inconsistency in the data and “intratechnique variability” [19].

### Amount of retraction:

Five out of the seven studies reported no significant difference in the amount of retraction of the active unit between the two methods; on the other hand, two studies [18, 23] reported a significant difference with more retraction of the maxillary incisors in the en masse group. This significance can be attributed to the fact that out of all the included studies both of these studies [18, 23] investigated class II cases with an overjet that is more than 5 mm while most of the other studies investigated patients with bi-maxillary proclination or class II cases with less severe overjet.

Data synthesis found a statistically significant difference in the amount of retraction between the two groups with a std mean difference of − 0.38 mm. This difference cannot be considered to be clinically significant. The standard mean difference detected between the two groups was represented by the distance between the tip of the UI and the SV vertical reference line which does not truly reflect the amount of bodily retraction of the UI but rather represents both bodily and angular changes in the anteroposterior direction. The different mechanics and archwires used by different operators could easily influence these results. This finding was surprising as there was a 2.5-mm difference in the amount of anchorage loss between the two methods. This might lead to an expectation of more retraction in the en masse/miniscrew group. Another aspect was that the cases included in these studies were either diagnosed with class II division 1 or bi-maxillary proclination. In the bi-maxillary proclination cases, even though extractions were needed, most of the space is used to correct the inclination rather than the position of the UI. Furthermore, the amount of retraction of the upper arch is limited by the position of the lower anterior teeth. The amount of retraction of the anterior teeth in the lower arch controls that of the upper regardless of the space available in the upper.

### Duration of treatment/retraction:

Five included studies reported either the duration of retraction or the duration of the overall treatment. Only two of the five studies [18, 24] found a significant difference with a shorter duration of treatment and retraction respectively in the en masse group. The other three studies found no significant difference in the overall treatment time nor the duration of retraction between the two groups. They suspected that this might be the result of the absence of any mesial movement of the posterior teeth in the en masse/miniscrews. This in turn might have led to complete closure of the space by the distal movement of the anterior segment. On the other hand, in the two-step/conventional anchorage group, the space closure is achieved by the simultaneous mesial movement of the posterior teeth and the distal movement of the anterior teeth.

### Root resorption

Two of the included studies [19, 24] reported on the amount of apical root resorption. Both studies reported no significant difference between the two methods of retraction. These two studies were found to be of low quality. A quantitative synthesis was not possible due to the difference in the measurement methods between the studies.

### Limitations

Articles published in languages other than English were excluded. This might have led to some potentially eligible studies to not be included in this review. Heterogeneity existed in the interventions as it was impossible to eliminate differences in the anchorage methods, archwire diameter, bracket prescription, and auxiliaries used. The differences in the outcome measurement methods were addressed by accepting minor methodological differences such as using different reference points to measure the cephalometric readings as long as the same tooth movement was being measured. Three of the included studies were judged to be of high risk of bias.

### Recommendations

The utilization of en masse retraction aided with the use of miniscrews as anchorage reinforcement is recommended in maximum anchorage cases. This recommendation is derived from the fact that this maximum anchorage space closure protocol was proven to be superior to other methods and combinations. This protocol showed better anchorage preservation and a shorter treatment time. This review found that high-quality studies are lacking and further well-designed RCTs are needed.

## Conclusions

- Both en masse and two-step retraction are effective methods in orthodontic space closure.
- En masse retraction is superior in anchorage preservation and incisor retraction if used in conjunction with miniscrews when compared to two-step retraction combined with conventional anchorage methods.
- Available evidence suggests that utilizing a headgear with both retraction methods produces no significant differences in the amount of incisor retraction or anchorage loss.
- Limited evidence suggests that the en masse method results in faster treatment.
- Limited low-quality evidence suggests that the amount of root resorption is not affected by the method of retraction
- Further high-quality studies directly comparing the two retraction techniques are needed.

## Additional file

Additional file 1: Table S1. MeSH terms and keywords used for the electronic database search. (DOCX 12 kb)

## Abbreviations

CI: Confidence intervals
pCCT: Prospective controlled clinical trial
RCT: Randomized clinical trial
RR: Root resorption
Std: Standardized
U6: Maxillary permanent first molar
UI: Maxillary permanent central incisor
