# Supplementary material for: Effectiveness of en masse versus two-step retraction: a systematic review and meta-analysis
Source: Prog Orthod. 2018 Jan 5;18:41. doi: 10.1186/s40510-017-0196-7 (PMC5754281; doi:10.1186/s40510-017-0196-7)
Supplement: Additional file 1: Table S1. — MeSH terms and keywords used for the electronic database search. (DOCX 12 kb) [file 40510_2017_196_MOESM1_ESM.docx]

| **Intervention** | **Comparison** | **MeSH terms** | **Others** |
| --- | --- | --- | --- |
| Enmasse | Two-step | "Orthodontic Anchorage Procedures"[Mesh] | Anchorage |
| En-masse | Canine retraction | "Root Resorption"[Mesh] | Tooth movement |
| En masse | 2 step | "Tooth Root"[Mesh] | Root resorption |
| On step retraction | 2 step retraction | "Tooth Movement"[Mesh] | Space closure |
| One step | Two step retraction | "Orthodontic Space Closure"  [Mesh] |  |
| One-step | Two step |  |  |

Supplementary Table 1 MeSH terms and keywords used for the electronic database search:
